# *N*-Ethyl-4-methyl-*N*-(3-methyl­phen­yl)benzene­sulfonamide

**DOI:** 10.1107/S160053681102589X

**Published:** 2011-07-06

**Authors:** Saba Ahmad, Muhammad Akhyar Farrukh, Fahim Ashraf Qureshi, Komal Faryal, Mehmet Akkurt

**Affiliations:** a Department of Chemistry, Government College University, Lahore 54000, Pakistan; b Department of Physics, Faculty of Sciences, Erciyes University, 38039 Kayseri, Turkey

## Abstract

The title compound, C_16_H_19_NO_2_S, crystallizes with two crystallographically independent mol­ecules in the asymmetric unit in which the dihedral angles between the planes defined by the aromatic rings are 35.3 (2) and 42.5 (2)°. In the crystal, inter­molecular C—H⋯O hydrogen bonds stabilize the packing.

## Related literature

For medicinal and pharmacological uses of sulfonamides, see: Betts *et al.* (2003 ▶); Brown (2000 ▶); Collery *et al.* (2008 ▶); Jones *et al.* (1997 ▶); Smolin *et al.* (1994 ▶). For related structures, see: Ahmad *et al.* (2011 ▶); Aziz-ur-Rehman *et al.* (2010*a* ▶,*b* ▶,*c* ▶); Khan *et al.* (2010 ▶).
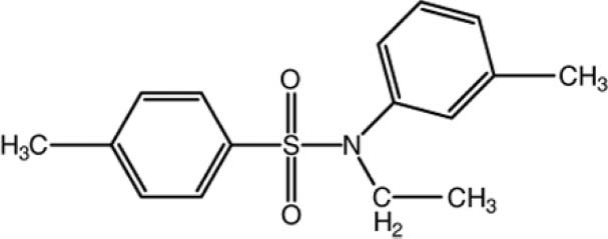


## Experimental

### 

#### Crystal data

C_16_H_19_NO_2_S
*M* *_r_* = 289.39
Monoclinic, 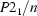

*a* = 19.753 (1) Å
*b* = 8.335 (1) Å
*c* = 20.395 (2) Å
β = 108.657 (3)°
*V* = 3181.4 (4) Å^3^
*Z* = 8
Mo *K*α radiation
μ = 0.20 mm^−1^
*T* = 296 K
0.25 × 0.13 × 0.09 mm

#### Data collection

Bruker APEXII CCD diffractometer
24592 measured reflections
5905 independent reflections
2779 reflections with *I* > 2σ(*I*)
*R*_int_ = 0.087

#### Refinement

*R*[*F* ^2^ > 2σ(*F* ^2^)] = 0.060
*wR*(*F* ^2^) = 0.173
*S* = 0.99
5905 reflections
366 parameters
H-atom parameters constrained
Δρ_max_ = 0.37 e Å^−3^
Δρ_min_ = −0.32 e Å^−3^

### 

Data collection: *APEX2* (Bruker, 2007 ▶); cell refinement: *SAINT* (Bruker, 2007 ▶); data reduction: *SAINT*; program(s) used to solve structure: *SIR97* (Altomare *et al.*, 1999 ▶); program(s) used to refine structure: *SHELXL97* (Sheldrick, 2008 ▶); molecular graphics: *ORTEP-3 for Windows* (Farrugia, 1997 ▶); software used to prepare material for publication: *WinGX* (Farrugia, 1999 ▶) and *PLATON* (Spek, 2009 ▶).

## Supplementary Material

Crystal structure: contains datablock(s) global, I. DOI: 10.1107/S160053681102589X/bt5567sup1.cif

Structure factors: contains datablock(s) I. DOI: 10.1107/S160053681102589X/bt5567Isup2.hkl

Supplementary material file. DOI: 10.1107/S160053681102589X/bt5567Isup3.cml

Additional supplementary materials:  crystallographic information; 3D view; checkCIF report

## Figures and Tables

**Table 1:** Hydrogen-bond geometry (Å, °)

*D*—H⋯*A*	*D*—H	H⋯*A*	*D*⋯*A*	*D*—H⋯*A*
C11—H11⋯O1^i^	0.93	2.48	3.398 (5)	171
C19—H19⋯O3^ii^	0.93	2.59	3.480 (5)	161
C27—H27⋯O4^iii^	0.93	2.57	3.503 (5)	176

Symmetry codes: (i) 
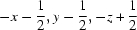
; (ii) 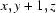; (iii) 
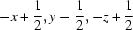
.

**Fig. 1.**
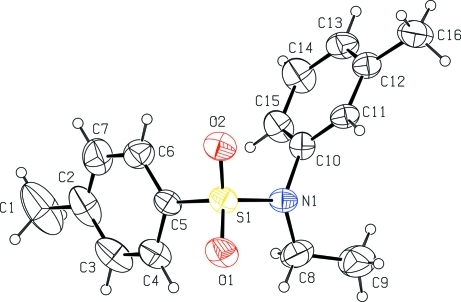
One of the two molecules of the title compound in the asymmetric unit. Displacement ellipsoids for non-H atoms are drawn at the 30% probability level.

**Fig. 2.**
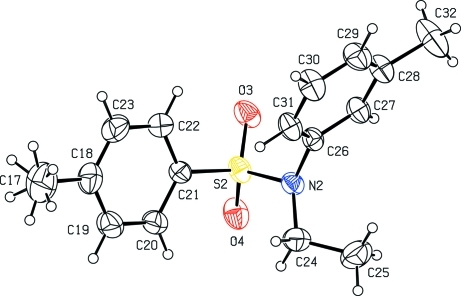
The other one of the two molecules of the title compound in the asymmetric unit. Displacement ellipsoids for non-H atoms are drawn at the 30% probability level.

**Fig. 3.**
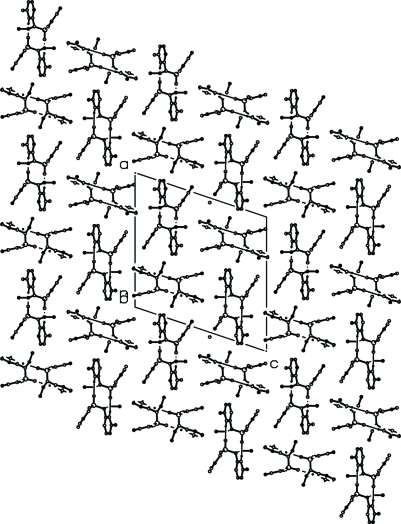
View of the crystal packing and the hydrogen bonding interactions of the title compound down the b axis. H atoms not involved in hydrogen bonds (dashed lines) have been omitted for clarity.

## supplementary crystallographic information

### Comment

Sulfonamides are well known compounds for medicinal and pharmacological uses. They are one of the important discoveries in medicinal sciences and has provided a new path for chemotherapy (Smolin *et al.*, 1994). N-substituted sulfonamides are widely used as anticancer, diuretic, hypoglycemic, anti-carbonic anhydrase, antithyroid and protease inhibitor agents (Collery *et al.*, 2008). Sulfonamides are also used in veterinary and fish pharmacology (Jones *et al.*, 1997 & Brown, 2000). They have bacteriostatic properties and are effective systematic drug used for humans (Betts *et al.*, 2003).

As a contribution to a structural study of sulfonamide derivatives (Khan *et al.*, 2010; Aziz-ur-Rehman *et al.*, 2010*a*,*b*,*c*, Ahmad *et al.*, 2011), we report here the title compound, *N*-ethyl-4-methyl-*N*-(3-methylphenyl)benzenesulfonamide, (I).

In each molecule of (I) in the asymmetric unit, (Fig. 1 and 2), the S atom has a distorted tetrahedral geometry [maximum deviation: O1—S1—O2 = 119.31 (18) ° for molecule A with S1 and O3—S2—O4 = 119.60 (17) ° for molecule B with S2]. The molecule A is twisted at the S atom, with a C5—S1—N1—C10 torsion angle of 80.2 (3) ° and the molecule B with a C21—S2—N2—C26 torsion angle of -79.4 (3) °. The dihedral angle formed between the benzene rings in (I) is 35.3 (2) ° for molecule A and is and 42.5 (2)° for molecule B.

The crystal structure is stabilized by intermolecular C—H···O hydrogen bonds (Table 1, Fig. 3).

### Experimental

5 m*M* of *m*-toluidine was dissolved in 20 ml of distilled water then 5 m*M* of ethyl iodide was added. The reaction mixture was stirred properly and 5 m*M* of *p*-toluenesulfonyl chloride was added. The mixture was stirred for about 1–2 h and the pH was maintained 8–10 using Na_2_CO_3_ solution 3%. The reaction was monitored by TLC. The product obtained was filtered and the precipitate was washed with distilled water, dried and recrystallized using methanol.

### Refinement

All H atoms were positioned geometrically with C—H = 0.93, 0.97 and 0.96 Å for aromatic, methylene and methyl H, respectively and treated using a riding model, with *U*_iso_(H) = 1.5*U*_eq_(C) for methyl H and *U*_iso_(H) = 1.2*U*_eq_(C) for all other H atoms. The H atoms of the methyl group C17 were refined as idealized disordered groups with two positions rotated from each other by 60°.

### Crystal data

**Table:**

C_16_H_19_NO_2_S	*F*(000) = 1232
*M**_r_* = 289.39	*D*_x_ = 1.208 Mg m^−^^3^
Monoclinic, *P*2_1_/*n*	Mo *K*α radiation, λ = 0.71073 Å
Hall symbol: -P 2yn	Cell parameters from 2536 reflections
*a* = 19.753 (1) Å	θ = 3.1–19.5°
*b* = 8.335 (1) Å	µ = 0.20 mm^−^^1^
*c* = 20.395 (2) Å	*T* = 296 K
β = 108.657 (3)°	Needle, colourless
*V* = 3181.4 (4) Å^3^	0.25 × 0.13 × 0.09 mm
*Z* = 8	

### Data collection

**Table:**

Bruker APEXII CCD diffractometer	2779 reflections with *I* > 2σ(*I*)
Radiation source: sealed tube	*R*_int_ = 0.087
graphite	θ_max_ = 25.5°, θ_min_ = 1.3°
φ and ω scans	*h* = −23→23
24592 measured reflections	*k* = −8→10
5905 independent reflections	*l* = −24→24

### Refinement

**Table:**

Refinement on *F*^2^	Primary atom site location: structure-invariant direct methods
Least-squares matrix: full	Secondary atom site location: difference Fourier map
*R*[*F*^2^ > 2σ(*F*^2^)] = 0.060	Hydrogen site location: inferred from neighbouring sites
*wR*(*F*^2^) = 0.173	H-atom parameters constrained
*S* = 0.99	*w* = 1/[σ^2^(*F*_o_^2^) + (0.073*P*)^2^] where *P* = (*F*_o_^2^ + 2*F*_c_^2^)/3
5905 reflections	(Δ/σ)_max_ < 0.001
366 parameters	Δρ_max_ = 0.37 e Å^−^^3^
0 restraints	Δρ_min_ = −0.32 e Å^−^^3^

### Special details

**Table:**

Geometry. Bond distances, angles *etc*. have been calculated using the rounded fractional coordinates. All su's are estimated from the variances of the (full) variance-covariance matrix. The cell e.s.d.'s are taken into account in the estimation of distances, angles and torsion angles
Refinement. Refinement on *F*^2^ for ALL reflections except those flagged by the user for potential systematic errors. Weighted *R*-factors *wR* and all goodnesses of fit *S* are based on *F*^2^, conventional *R*-factors *R* are based on *F*, with *F* set to zero for negative *F*^2^. The observed criterion of *F*^2^ > σ(*F*^2^) is used only for calculating -*R*-factor-obs *etc*. and is not relevant to the choice of reflections for refinement. *R*-factors based on *F*^2^ are statistically about twice as large as those based on *F*, and *R*-factors based on ALL data will be even larger.

### Fractional atomic coordinates and isotropic or equivalent isotropic displacement parameters (Å^2^)

**Table:**

	*x*	*y*	*z*	*U*_iso_*/*U*_eq_	Occ. (<1)
S2	0.30839 (5)	0.21131 (11)	0.33137 (6)	0.0586 (4)	
O3	0.34832 (13)	0.0725 (3)	0.36279 (15)	0.0732 (12)	
O4	0.30175 (14)	0.2502 (3)	0.26194 (15)	0.0764 (11)	
N2	0.22738 (14)	0.1862 (3)	0.33484 (16)	0.0528 (10)	
C17	0.4182 (3)	0.7947 (6)	0.5085 (3)	0.130 (3)	
C18	0.3931 (2)	0.6476 (6)	0.4640 (3)	0.0804 (19)	
C19	0.3549 (2)	0.6621 (5)	0.3957 (3)	0.0792 (19)	
C20	0.3303 (2)	0.5302 (5)	0.3542 (2)	0.0686 (17)	
C21	0.34449 (18)	0.3789 (4)	0.3824 (2)	0.0549 (14)	
C22	0.3833 (2)	0.3611 (5)	0.4506 (2)	0.0735 (19)	
C23	0.4077 (2)	0.4953 (6)	0.4911 (2)	0.0874 (19)	
C24	0.17367 (19)	0.3093 (5)	0.3030 (2)	0.0706 (17)	
C25	0.0997 (2)	0.2383 (6)	0.2796 (3)	0.100 (2)	
C26	0.22070 (16)	0.1089 (4)	0.3956 (2)	0.0492 (14)	
C27	0.20519 (18)	−0.0524 (4)	0.3917 (2)	0.0587 (14)	
C28	0.1949 (2)	−0.1317 (5)	0.4468 (2)	0.0715 (19)	
C29	0.2020 (2)	−0.0471 (5)	0.5064 (2)	0.0761 (17)	
C30	0.2180 (2)	0.1138 (5)	0.5109 (2)	0.0770 (17)	
C31	0.2264 (2)	0.1913 (4)	0.4553 (2)	0.0634 (16)	
C32	0.1764 (3)	−0.3090 (5)	0.4404 (3)	0.127 (3)	
S1	−0.16509 (5)	−0.14051 (12)	0.31425 (6)	0.0648 (4)	
O1	−0.23579 (13)	−0.0975 (3)	0.31230 (16)	0.0877 (13)	
O2	−0.13463 (14)	−0.2857 (3)	0.34707 (15)	0.0786 (11)	
N1	−0.16606 (15)	−0.1498 (4)	0.23453 (17)	0.0619 (13)	
C1	0.0351 (3)	0.4102 (8)	0.4320 (4)	0.190 (4)	
C2	−0.0154 (4)	0.2711 (8)	0.4058 (4)	0.111 (3)	
C3	−0.0847 (4)	0.2978 (6)	0.3656 (3)	0.111 (3)	
C4	−0.1314 (2)	0.1734 (5)	0.3393 (3)	0.0856 (19)	
C5	−0.1080 (2)	0.0180 (5)	0.3528 (2)	0.0634 (17)	
C6	−0.0396 (2)	−0.0099 (6)	0.3931 (2)	0.0838 (19)	
C7	0.0056 (3)	0.1178 (8)	0.4193 (3)	0.108 (3)	
C8	−0.1985 (3)	−0.0114 (6)	0.1890 (3)	0.105 (2)	
C9	−0.2221 (3)	−0.0469 (7)	0.1165 (3)	0.130 (3)	
C10	−0.10618 (19)	−0.2300 (5)	0.2214 (2)	0.0613 (16)	
C11	−0.11588 (18)	−0.3812 (5)	0.1959 (2)	0.0623 (16)	
C12	−0.0620 (2)	−0.4620 (5)	0.1791 (2)	0.0683 (16)	
C13	0.0024 (2)	−0.3826 (6)	0.1910 (2)	0.0801 (19)	
C14	0.0117 (2)	−0.2301 (6)	0.2176 (3)	0.098 (2)	
C15	−0.0421 (2)	−0.1498 (5)	0.2321 (2)	0.0819 (19)	
C16	−0.0740 (2)	−0.6272 (5)	0.1484 (3)	0.105 (2)	
H17A	0.44420	0.76230	0.55490	0.1960*	0.500
H17B	0.37760	0.85780	0.50870	0.1960*	0.500
H17C	0.44870	0.85740	0.49010	0.1960*	0.500
H17D	0.40280	0.88940	0.48090	0.1960*	0.500
H17E	0.46940	0.79390	0.52710	0.1960*	0.500
H17F	0.39830	0.79430	0.54570	0.1960*	0.500
H19	0.34520	0.76400	0.37640	0.0950*	
H20	0.30420	0.54320	0.30770	0.0820*	
H22	0.39320	0.25910	0.46980	0.0880*	
H23	0.43450	0.48260	0.53750	0.1050*	
H24A	0.18390	0.35580	0.26360	0.0850*	
H24B	0.17610	0.39410	0.33620	0.0850*	
H25A	0.09640	0.15890	0.24470	0.1500*	
H25B	0.06530	0.32150	0.26100	0.1500*	
H25C	0.09010	0.18930	0.31830	0.1500*	
H27	0.20160	−0.10850	0.35130	0.0700*	
H29	0.19580	−0.09950	0.54430	0.0910*	
H30	0.22310	0.16940	0.55170	0.0920*	
H31	0.23610	0.30080	0.45780	0.0760*	
H32A	0.21940	−0.37110	0.45770	0.1900*	
H32B	0.15280	−0.33510	0.39270	0.1900*	
H32C	0.14520	−0.33280	0.46690	0.1900*	
H1A	0.04570	0.45970	0.39390	0.2840*	
H1B	0.07860	0.37190	0.46500	0.2840*	
H1C	0.01320	0.48750	0.45380	0.2840*	
H3	−0.10060	0.40280	0.35570	0.1320*	
H4	−0.17840	0.19440	0.31270	0.1030*	
H6	−0.02330	−0.11460	0.40290	0.1010*	
H7	0.05220	0.09690	0.44730	0.1300*	
H8A	−0.23900	0.02740	0.20160	0.1250*	
H8B	−0.16370	0.07460	0.19760	0.1250*	
H9A	−0.18240	−0.08390	0.10320	0.1950*	
H9B	−0.24170	0.04820	0.09090	0.1950*	
H9C	−0.25820	−0.12880	0.10690	0.1950*	
H11	−0.15940	−0.43210	0.18950	0.0750*	
H13	0.03980	−0.43340	0.18080	0.0960*	
H14	0.05580	−0.18000	0.22590	0.1180*	
H15	−0.03590	−0.04500	0.24870	0.0980*	
H16A	−0.03600	−0.65380	0.13050	0.1570*	
H16B	−0.11880	−0.63020	0.11170	0.1570*	
H16C	−0.07480	−0.70320	0.18350	0.1570*	

### Atomic displacement parameters (Å^2^)

**Table:**

	*U*^11^	*U*^22^	*U*^33^	*U*^12^	*U*^13^	*U*^23^
S2	0.0635 (6)	0.0536 (6)	0.0688 (8)	−0.0062 (5)	0.0353 (5)	−0.0012 (5)
O3	0.0685 (16)	0.0485 (15)	0.114 (3)	0.0114 (12)	0.0454 (16)	0.0103 (15)
O4	0.095 (2)	0.0828 (19)	0.064 (2)	−0.0249 (14)	0.0430 (16)	−0.0084 (15)
N2	0.0544 (17)	0.0501 (17)	0.058 (2)	−0.0003 (14)	0.0239 (15)	0.0074 (15)
C17	0.142 (5)	0.115 (4)	0.122 (5)	−0.030 (4)	0.025 (4)	−0.050 (4)
C18	0.080 (3)	0.082 (3)	0.077 (4)	−0.019 (2)	0.022 (3)	−0.017 (3)
C19	0.097 (3)	0.052 (3)	0.081 (4)	−0.004 (2)	0.018 (3)	−0.004 (3)
C20	0.079 (3)	0.062 (3)	0.059 (3)	−0.005 (2)	0.014 (2)	0.008 (2)
C21	0.054 (2)	0.056 (2)	0.055 (3)	−0.0028 (17)	0.018 (2)	0.010 (2)
C22	0.069 (3)	0.071 (3)	0.071 (4)	−0.004 (2)	0.009 (2)	0.013 (3)
C23	0.083 (3)	0.106 (4)	0.059 (3)	−0.014 (3)	0.003 (2)	0.003 (3)
C24	0.071 (3)	0.069 (3)	0.070 (3)	0.010 (2)	0.020 (2)	0.012 (2)
C25	0.063 (3)	0.127 (4)	0.101 (4)	0.010 (3)	0.013 (3)	0.020 (3)
C26	0.048 (2)	0.046 (2)	0.059 (3)	−0.0017 (16)	0.0247 (19)	0.0014 (19)
C27	0.070 (2)	0.049 (2)	0.064 (3)	−0.0039 (18)	0.031 (2)	−0.006 (2)
C28	0.086 (3)	0.057 (3)	0.079 (4)	−0.012 (2)	0.037 (3)	0.002 (2)
C29	0.095 (3)	0.080 (3)	0.063 (3)	−0.007 (2)	0.039 (3)	0.010 (3)
C30	0.105 (3)	0.071 (3)	0.068 (3)	−0.017 (2)	0.046 (3)	−0.014 (2)
C31	0.077 (3)	0.048 (2)	0.075 (3)	−0.0064 (18)	0.038 (2)	−0.008 (2)
C32	0.203 (6)	0.071 (3)	0.127 (5)	−0.042 (3)	0.082 (5)	−0.003 (3)
S1	0.0591 (6)	0.0658 (7)	0.0777 (8)	−0.0024 (5)	0.0335 (6)	0.0005 (6)
O1	0.0584 (16)	0.106 (2)	0.113 (3)	−0.0004 (14)	0.0473 (17)	−0.0134 (18)
O2	0.0895 (19)	0.0627 (17)	0.092 (2)	0.0016 (15)	0.0407 (17)	0.0167 (16)
N1	0.0506 (17)	0.067 (2)	0.073 (3)	0.0053 (15)	0.0268 (17)	0.0043 (18)
C1	0.151 (5)	0.164 (6)	0.299 (10)	−0.094 (5)	0.136 (6)	−0.138 (6)
C2	0.103 (4)	0.108 (5)	0.151 (6)	−0.043 (4)	0.080 (4)	−0.061 (4)
C3	0.132 (5)	0.070 (3)	0.157 (6)	−0.005 (3)	0.085 (5)	−0.017 (3)
C4	0.079 (3)	0.071 (3)	0.116 (4)	−0.003 (3)	0.044 (3)	−0.011 (3)
C5	0.062 (3)	0.064 (3)	0.073 (3)	0.0031 (19)	0.034 (2)	−0.002 (2)
C6	0.068 (3)	0.091 (3)	0.092 (4)	0.000 (3)	0.025 (3)	−0.001 (3)
C7	0.071 (3)	0.141 (5)	0.114 (5)	−0.022 (4)	0.031 (3)	−0.042 (4)
C8	0.089 (3)	0.133 (5)	0.083 (4)	0.036 (3)	0.015 (3)	0.015 (4)
C9	0.150 (5)	0.141 (5)	0.099 (5)	0.052 (4)	0.040 (4)	0.031 (4)
C10	0.057 (2)	0.069 (3)	0.062 (3)	−0.0093 (19)	0.025 (2)	−0.003 (2)
C11	0.046 (2)	0.065 (3)	0.075 (3)	0.0003 (19)	0.018 (2)	0.008 (2)
C12	0.060 (2)	0.069 (3)	0.076 (3)	0.001 (2)	0.022 (2)	0.004 (2)
C13	0.058 (3)	0.097 (3)	0.093 (4)	0.009 (2)	0.035 (2)	0.009 (3)
C14	0.064 (3)	0.109 (4)	0.135 (5)	−0.029 (3)	0.053 (3)	−0.029 (3)
C15	0.065 (3)	0.084 (3)	0.107 (4)	−0.021 (2)	0.042 (3)	−0.024 (3)
C16	0.080 (3)	0.084 (3)	0.142 (5)	0.014 (2)	0.025 (3)	−0.012 (3)

### Geometric parameters (Å, °)

**Table:**

S2—O3	1.431 (3)	C27—H27	0.9300
S2—O4	1.417 (3)	C29—H29	0.9300
S2—N2	1.638 (3)	C30—H30	0.9300
S2—C21	1.752 (4)	C31—H31	0.9300
S1—N1	1.622 (3)	C32—H32C	0.9600
S1—O1	1.430 (3)	C32—H32A	0.9600
S1—O2	1.420 (3)	C32—H32B	0.9600
S1—C5	1.752 (4)	C1—C2	1.511 (10)
N2—C24	1.469 (5)	C2—C3	1.370 (11)
N2—C26	1.440 (5)	C2—C7	1.344 (9)
N1—C10	1.456 (5)	C3—C4	1.377 (8)
N1—C8	1.491 (6)	C4—C5	1.373 (6)
C17—C18	1.512 (7)	C5—C6	1.358 (6)
C18—C19	1.361 (8)	C6—C7	1.382 (8)
C18—C23	1.378 (7)	C8—C9	1.432 (8)
C19—C20	1.378 (6)	C10—C11	1.353 (6)
C20—C21	1.377 (5)	C10—C15	1.386 (6)
C21—C22	1.365 (5)	C11—C12	1.392 (6)
C22—C23	1.382 (6)	C12—C13	1.385 (6)
C24—C25	1.506 (6)	C12—C16	1.500 (6)
C26—C27	1.376 (5)	C13—C14	1.371 (7)
C26—C31	1.371 (5)	C14—C15	1.366 (6)
C27—C28	1.374 (5)	C1—H1A	0.9600
C28—C29	1.373 (6)	C1—H1B	0.9600
C28—C32	1.518 (6)	C1—H1C	0.9600
C29—C30	1.374 (6)	C3—H3	0.9300
C30—C31	1.361 (6)	C4—H4	0.9300
C17—H17C	0.9600	C6—H6	0.9300
C17—H17A	0.9600	C7—H7	0.9300
C17—H17B	0.9600	C8—H8A	0.9700
C17—H17F	0.9600	C8—H8B	0.9700
C17—H17D	0.9600	C9—H9A	0.9600
C17—H17E	0.9600	C9—H9B	0.9600
C19—H19	0.9300	C9—H9C	0.9600
C20—H20	0.9300	C11—H11	0.9300
C22—H22	0.9300	C13—H13	0.9300
C23—H23	0.9300	C14—H14	0.9300
C24—H24B	0.9700	C15—H15	0.9300
C24—H24A	0.9700	C16—H16A	0.9600
C25—H25C	0.9600	C16—H16B	0.9600
C25—H25B	0.9600	C16—H16C	0.9600
C25—H25A	0.9600		
			
O3—S2—O4	119.60 (17)	H25A—C25—H25B	110.00
O3—S2—N2	106.47 (15)	H25B—C25—H25C	109.00
O3—S2—C21	108.51 (18)	C28—C27—H27	120.00
O4—S2—N2	107.04 (17)	C26—C27—H27	120.00
O4—S2—C21	107.53 (17)	C28—C29—H29	119.00
N2—S2—C21	107.09 (17)	C30—C29—H29	119.00
O1—S1—O2	119.31 (18)	C29—C30—H30	120.00
O1—S1—N1	106.61 (18)	C31—C30—H30	120.00
O1—S1—C5	107.83 (18)	C30—C31—H31	120.00
O2—S1—N1	107.41 (18)	C26—C31—H31	120.00
O2—S1—C5	108.44 (19)	C28—C32—H32B	109.00
N1—S1—C5	106.59 (18)	C28—C32—H32C	109.00
S2—N2—C26	117.0 (2)	H32A—C32—H32C	109.00
C24—N2—C26	116.2 (3)	H32B—C32—H32C	109.00
S2—N2—C24	118.0 (2)	H32A—C32—H32B	110.00
S1—N1—C8	116.9 (3)	C28—C32—H32A	109.00
S1—N1—C10	117.2 (3)	C1—C2—C3	120.5 (6)
C8—N1—C10	117.0 (4)	C1—C2—C7	122.1 (7)
C17—C18—C19	120.7 (4)	C3—C2—C7	117.4 (6)
C19—C18—C23	118.0 (4)	C2—C3—C4	121.8 (5)
C17—C18—C23	121.3 (5)	C3—C4—C5	119.4 (5)
C18—C19—C20	121.9 (4)	S1—C5—C4	119.6 (3)
C19—C20—C21	119.3 (4)	S1—C5—C6	121.0 (3)
C20—C21—C22	119.9 (3)	C4—C5—C6	119.3 (4)
S2—C21—C22	120.5 (3)	C5—C6—C7	119.8 (5)
S2—C21—C20	119.5 (3)	C2—C7—C6	122.3 (6)
C21—C22—C23	119.7 (4)	N1—C8—C9	114.6 (4)
C18—C23—C22	121.2 (4)	N1—C10—C11	118.3 (3)
N2—C24—C25	110.9 (3)	N1—C10—C15	120.3 (4)
N2—C26—C27	117.9 (3)	C11—C10—C15	121.3 (4)
N2—C26—C31	122.4 (3)	C10—C11—C12	121.3 (4)
C27—C26—C31	119.6 (3)	C11—C12—C13	117.2 (4)
C26—C27—C28	120.8 (4)	C11—C12—C16	121.1 (4)
C29—C28—C32	122.0 (4)	C13—C12—C16	121.7 (4)
C27—C28—C29	118.5 (4)	C12—C13—C14	120.8 (4)
C27—C28—C32	119.5 (4)	C13—C14—C15	121.7 (4)
C28—C29—C30	121.1 (4)	C10—C15—C14	117.7 (4)
C29—C30—C31	119.6 (4)	C2—C1—H1A	109.00
C26—C31—C30	120.3 (3)	C2—C1—H1B	110.00
C18—C17—H17C	109.00	C2—C1—H1C	110.00
C18—C17—H17D	109.00	H1A—C1—H1B	109.00
C18—C17—H17E	109.00	H1A—C1—H1C	109.00
C18—C17—H17F	109.00	H1B—C1—H1C	109.00
H17A—C17—H17B	110.00	C2—C3—H3	119.00
H17A—C17—H17C	110.00	C4—C3—H3	119.00
C18—C17—H17B	109.00	C3—C4—H4	120.00
H17C—C17—H17E	56.00	C5—C4—H4	120.00
H17C—C17—H17F	141.00	C5—C6—H6	120.00
H17D—C17—H17E	109.00	C7—C6—H6	120.00
H17D—C17—H17F	109.00	C2—C7—H7	119.00
H17E—C17—H17F	109.00	C6—C7—H7	119.00
H17B—C17—H17E	141.00	N1—C8—H8A	109.00
H17B—C17—H17F	56.00	N1—C8—H8B	109.00
H17A—C17—H17D	141.00	C9—C8—H8A	109.00
H17A—C17—H17E	56.00	C9—C8—H8B	109.00
H17A—C17—H17F	56.00	H8A—C8—H8B	108.00
H17B—C17—H17C	109.00	C8—C9—H9A	109.00
H17B—C17—H17D	56.00	C8—C9—H9B	109.00
C18—C17—H17A	109.00	C8—C9—H9C	109.00
H17C—C17—H17D	56.00	H9A—C9—H9B	110.00
C20—C19—H19	119.00	H9A—C9—H9C	110.00
C18—C19—H19	119.00	H9B—C9—H9C	109.00
C19—C20—H20	120.00	C10—C11—H11	119.00
C21—C20—H20	120.00	C12—C11—H11	119.00
C21—C22—H22	120.00	C12—C13—H13	120.00
C23—C22—H22	120.00	C14—C13—H13	120.00
C18—C23—H23	119.00	C13—C14—H14	119.00
C22—C23—H23	119.00	C15—C14—H14	119.00
N2—C24—H24A	109.00	C10—C15—H15	121.00
C25—C24—H24B	110.00	C14—C15—H15	121.00
H24A—C24—H24B	108.00	C12—C16—H16A	109.00
C25—C24—H24A	109.00	C12—C16—H16B	109.00
N2—C24—H24B	109.00	C12—C16—H16C	109.00
C24—C25—H25B	109.00	H16A—C16—H16B	110.00
C24—C25—H25A	110.00	H16A—C16—H16C	109.00
H25A—C25—H25C	109.00	H16B—C16—H16C	109.00
C24—C25—H25C	109.00		
			
O3—S2—N2—C24	−177.3 (3)	C19—C18—C23—C22	−1.1 (7)
O3—S2—N2—C26	36.5 (3)	C17—C18—C23—C22	178.6 (4)
O4—S2—N2—C24	−48.3 (3)	C18—C19—C20—C21	0.1 (7)
O4—S2—N2—C26	165.5 (2)	C19—C20—C21—S2	175.8 (3)
C21—S2—N2—C24	66.8 (3)	C19—C20—C21—C22	−0.8 (6)
C21—S2—N2—C26	−79.4 (3)	C20—C21—C22—C23	0.5 (6)
O3—S2—C21—C20	161.0 (3)	S2—C21—C22—C23	−176.1 (3)
O4—S2—C21—C20	30.3 (4)	C21—C22—C23—C18	0.5 (6)
N2—S2—C21—C20	−84.4 (3)	N2—C26—C27—C28	−177.0 (3)
O3—S2—C21—C22	−22.4 (4)	N2—C26—C31—C30	178.4 (4)
O4—S2—C21—C22	−153.1 (3)	C31—C26—C27—C28	0.4 (6)
N2—S2—C21—C22	92.1 (3)	C27—C26—C31—C30	1.1 (6)
C5—S1—N1—C10	80.2 (3)	C26—C27—C28—C32	178.8 (4)
O2—S1—C5—C4	−168.0 (4)	C26—C27—C28—C29	−1.4 (6)
O1—S1—N1—C8	48.9 (4)	C27—C28—C29—C30	0.9 (6)
O1—S1—N1—C10	−164.9 (3)	C32—C28—C29—C30	−179.3 (4)
O2—S1—N1—C8	177.9 (3)	C28—C29—C30—C31	0.6 (6)
O2—S1—N1—C10	−35.9 (3)	C29—C30—C31—C26	−1.6 (6)
C5—S1—N1—C8	−66.1 (4)	C1—C2—C3—C4	178.1 (6)
N1—S1—C5—C4	76.7 (4)	C7—C2—C3—C4	−0.5 (11)
O1—S1—C5—C4	−37.5 (4)	C1—C2—C7—C6	−177.3 (6)
O2—S1—C5—C6	15.4 (4)	C3—C2—C7—C6	1.3 (10)
N1—S1—C5—C6	−99.9 (4)	C2—C3—C4—C5	−0.8 (9)
O1—S1—C5—C6	145.9 (3)	C3—C4—C5—C6	1.4 (7)
C24—N2—C26—C31	−62.4 (5)	C3—C4—C5—S1	−175.3 (4)
S2—N2—C26—C31	84.4 (4)	C4—C5—C6—C7	−0.7 (7)
S2—N2—C26—C27	−98.2 (3)	S1—C5—C6—C7	175.9 (4)
S2—N2—C24—C25	152.5 (3)	C5—C6—C7—C2	−0.7 (8)
C26—N2—C24—C25	−61.0 (4)	N1—C10—C11—C12	176.9 (4)
C24—N2—C26—C27	115.0 (4)	C15—C10—C11—C12	−0.3 (6)
C10—N1—C8—C9	55.6 (6)	N1—C10—C15—C14	−178.7 (4)
C8—N1—C10—C15	65.9 (5)	C11—C10—C15—C14	−1.6 (6)
S1—N1—C8—C9	−158.1 (4)	C10—C11—C12—C13	1.4 (6)
C8—N1—C10—C11	−111.4 (4)	C10—C11—C12—C16	−177.7 (4)
S1—N1—C10—C15	−80.4 (4)	C11—C12—C13—C14	−0.6 (6)
S1—N1—C10—C11	102.4 (4)	C16—C12—C13—C14	178.5 (5)
C17—C18—C19—C20	−178.9 (5)	C12—C13—C14—C15	−1.3 (7)
C23—C18—C19—C20	0.8 (7)	C13—C14—C15—C10	2.3 (7)

### Hydrogen-bond geometry (Å, °)

**Table:**

*D*—H···*A*	*D*—H	H···*A*	*D*···*A*	*D*—H···*A*
C6—H6···O2	0.93	2.56	2.926 (5)	104
C8—H8A···O1	0.97	2.47	2.927 (7)	109
C11—H11···O1^i^	0.93	2.48	3.398 (5)	171
C19—H19···O3^ii^	0.93	2.59	3.480 (5)	161
C22—H22···O3	0.93	2.59	2.946 (5)	103
C24—H24A···O4	0.97	2.50	2.950 (5)	108
C27—H27···O4^iii^	0.93	2.57	3.503 (5)	176

Symmetry codes: (i) −*x*−1/2, *y*−1/2, −*z*+1/2; (ii) *x*, *y*+1, *z*; (iii) −*x*+1/2, *y*−1/2, −*z*+1/2.
